# Low prevalence of high-risk human papillomavirus in nail unit squamous cell carcinoma in Koreans using next-generation sequencing

**DOI:** 10.1016/j.jdin.2025.09.002

**Published:** 2025-09-25

**Authors:** Cheol Lee, Ji Su Lee, Sumi Yun, Kyung-Ah Hwang, Yoonjin Kwak, Je-Ho Mun

**Affiliations:** a Department of Pathology, Seoul National University Hospital, Seoul, Republic of Korea; b Department of Pathology, Seoul National University College of Medicine, Seoul, Republic of Korea; c Department of Dermatology, Seoul National University College of Medicine, Seoul, Republic of Korea; d Department of Dermatology, Seoul Metropolitan Government – Seoul National University (SMG-SNU) Boramae Medical Center, Seoul, Republic of Korea; e Institute of Human-Environmental Interface Biology, Medical Research Center, Seoul National University, Seoul, Republic of Korea; f Department of Pathology, Samkwang Medical Laboratories, Seoul, Republic of Korea; g Genomic Strategy Department, Samkwang Labtree, Seoul, Republic of Korea; h Department of Dermatology, Seoul National University Hospital, Seoul, Republic of Korea

**Keywords:** high-throughput nucleotide sequencing, HPV, human papillomavirus viruses, nails, nail unit squamous cell carcinoma, squamous cell carcinoma

*To the Editor:* Nail squamous cell carcinoma (NSCC) is a rare malignancy with multiple etiologies, including chronic trauma, immunosuppression, ultraviolet radiation, arsenic exposure, and human papillomavirus (HPV) infection.^1^ It was recently proposed that NSCC should be classified as a sexually transmitted disease (STD), citing its potential role as a reservoir for high-risk HPV.^2^ However, this classification could impose stigma and psychosocial distress on patients with NSCC.^3^ The data relied primarily on retrospective case series, which lack sufficient evidence to establish a causal link.^2^ Nail skin differs from genital mucosa, which is more susceptible to persistent HPV infection. Therefore, further studies are required.

We investigated the prevalence of HPV infections among Koreans with NSCC using next-generation sequencing (NGS). This study was approved by the Institutional Review Board. We analyzed formalin-fixed, paraffin-embedded tissue samples from NSCC cases from 2014 to 2022 at the Seoul National University Hospital. DNA extraction and HPV detection were performed using the Ezplex HPV NGS 100 Typing G Kit (SMLgenetree, Seoul, Korea), which detects 100 HPV subtypes, including types 1 to 45, 47 to 50, 51 to 63, 66 to 70, 71 to 78, 81 to 87, 89 to 95, 97, 99 to 103, 107 to 109, 114, and 120 (detailed Supplementary Methods available via Mendeley at https://data.mendeley.com/preview/jkj7x7pjkg).^4^

A total of 27 NSCC cases were analyzed (Table I). All tumors involved the nail bed or matrix, and none of them showed periungual-only (wart-type) involvement. The mean patient age was 58.2 ± 11.4 years (14 males and 13 females). The fingernails (74.1%) were more frequently affected than the toenails, with the first digit being the most common site (40.7%). Most tumors were invasive (85.2%). High-risk HPV, specifically HPV-51 and HPV-56, was detected in 2 cases (7.1%). However, HPV was not detected in the remaining 25 cases.

**Table I:** Demographic and clinical characteristics of patients with nail squamous cell carcinoma in Koreans and the results of next-generation sequencing of human papillomavirus

Patient no.	Sex	Age at diagnosis (y)	History of trauma	Single/multiple	Side	Location	Digit affected	Medical comorbidities including immunosuppression/cancer	Tumor type (in situ/invasive)	NGS analysis of HPV status
1	F	70	No	Single	Right	Finger	Fifth	Psoriasis, arsenical keratosis, Bowen's disease	Invasive	No
2	M	73	No	Single	Right	Toe	Fifth		Invasive	No
3	F	51	Yes	Single	Right	Finger	Fourth		Invasive	No
4	M	80	No	Single	Right	Finger	Second	Nonsmall cell lung carcinoma	In situ	HPV-51
5	M	59	No	Single	Right	Finger	Fourth	Nasopharyngeal cancer	Invasive	No
6	M	59	Yes	Single	Left	Finger	First	Diabetes, hypertension, dyslipidemia	Invasive	No
7	F	77	No	Single	Right	Finger	First	Diabetes, hypertension, dyslipidemia	Invasive	No
8	M	67	No	Single	Right	Finger	First		Invasive	No
9	M	60	Yes	Single	Left	Toe	Third	Diabetes	Invasive	No
10	M	78	No	Single	Left	Finger	Fourth		Invasive	No
11	F	49	No	Single	Right	Finger	First		Invasive	No
12	M	62	Yes	Single	Right	Finger	Second		Invasive	No
13	F	61	No	Single	Right	Finger	Third		Invasive	No
14	M	68	No	Single	Left	Finger	Third		Invasive	No
15	M	74	No	Single	Right	Finger	First	Acral melanoma, hypertension	In situ	No
16	M	61	No	Single	Left	Finger	Fourth	Multiple myeloma	Invasive	No
17	F	52	Yes	Single	Right	Toe	First	Crohn’s disease, breast cancer	In situ	No
18	F	61	Yes	Single	Right	Finger	Fourth		Invasive	No
19	M	67	Yes	Single	Left	Toe	Third		In situ	No
20	F	63	No	Single	Left	Finger	First		Invasive	No
21	F	76	No	Single	Left	Toe	Third	DM, hyperlipidemia	Invasive	No
22	F	45	No	Single	Right	Finger	First	Breast cancer	Invasive	No
23	F	65	No	Single	Left	Toe	First	Left breast cancer, tuberculosis	Invasive	No
24	F	72	No	Single	Right	Finger	Third	Dyslipidemia, hypertension, cervix HPV infection (HPV-33) (low-grade squamous epithelial lesion)	Invasive	HPV-56
25	M	59	Yes	Single	Left	Finger	First	Gastric cancer	Invasive	No
26	M	71	No	Single	Left	Toe	First	Melanoma, adrenal incidentaloma, hypertension, dyslipidemia, hypothyroidism	Invasive	No
27	F	58	Yes	Single	Right	Finger	Second	Diabetes, hypertension	Invasive	No

*DM*, Diabetes mellitus; *F*, female; *HPV*, human papillomavirus; *M*, male; *NGS*, next-generation sequencing.

The low prevalence of high-risk HPV is consistent with previous studies reporting the low detection of high-risk HPV in digital Bowen’s disease or SCC in Korean patients.^5^ Although HPV is detected in some SCCs and can be transmitted via skin contact, classifying NSCC as an STD is problematic due to limited evidence of sexual transmission. Such a classification may contribute to stigma, shame, and anxiety, thereby increasing the psychosocial burden on patients. Therefore, until larger studies provide stronger evidence, NSCC should not be categorized as an STD, but rather recognized as a multifactorial malignancy with diverse oncogenic etiologies.

Our study has limitations, including the small sample size and single-institution cohort. Additionally, periungual-type and polydactylous cases, which may have had different HPV associations, were not included. Our study focused on Korean patients. Geographic and ethnic variations in HPV prevalence and oncogenesis remain as possible influencing factors.

NGS offers advantages over conventional HPV detection methods, including higher sensitivity, broader genotype detection, and improved accuracy.^4^ Unlike polymerase chain reaction–based techniques, which often target a limited number of HPV types, NGS can detect rare and low-abundance HPV strains, minimizing false negatives.

Collectively, our NGS-based analysis revealed a low prevalence of high-risk HPV in NSCC. Future studies with larger and more diverse cohorts are required to clarify HPV’s role in NSCC pathogenesis.

## Conflicts of interest

None disclosed.
